# TEM1 up-regulates MMP-2 and promotes ECM remodeling for facilitating invasion and migration of uterine sarcoma

**DOI:** 10.1007/s12672-023-00613-6

**Published:** 2023-01-13

**Authors:** Chenghao Wu, Wenhuizi Sun, Dongsheng Shen, Huaifang Li, Xiaowen Tong, Yi Guo

**Affiliations:** Department of Obstetrics and Gynecology, Tongji Hospital, Tongji University School of Medicine, No.389 Xincun Road, Shanghai, 200065 People’s Republic of China

**Keywords:** Tumor endothelial marker 1/TEM1, Uterine leiomyosarcoma, Matrix metalloproteinase 2/MMP-2, Metastasis, Invasion, Migration

## Abstract

**Objectives:**

To explore the correlation between tumor endothelial marker 1 (TEM1) and matrix metalloproteinase 2 (MMP-2) in uterine sarcoma and their roles in the progression of uterine sarcoma.

**Methods:**

Uterine leiomyosarcoma (uLMS, n = 25) and uterine leiomyoma (n = 25) specimens were collected from a total of 50 patients. Immunohistochemistry assay was conducted to determine the expression of TEM1, MMP-2 and MMP-9. TEM1 over expression (hTEM1) and low expression (shRNA-TEM1) MES-SA cell lines were established as in vitro uterine sarcoma models. MMP-2 mRNA, protein expression and enzymatic activity were verified using qPCR, Western blot and gelatin zymography respectively. MMP-2 expression was downregulated using MMP-2 siRNA in hTEM1 MES-SA cells to better study the role of MMP-2. The invasive and migratory capacities of hTEM1, shRNA-TEM1, and hTEM1 treated with MMP-2 siRNA MES-SA cells were determined using transwell assays. Extracellular matrix (ECM) remodeling mediated by TEM1 was examined using cell-ECM adhesion and fluorescent gelatin-ECM degradation assays. The immunofluorescence of F-actin was examined to analyze the formation of invadopodia. Subcutaneous and intraperitoneal xenografts were established to validate the role of TEM1 in promoting uterine sarcoma metastasis.

**Results:**

TEM1 and MMP-2 were expressed in 92% (n = 23) and 88% (n = 22) of uterine leiomyosarcoma specimens, respectively. Both TEM1 and MMP-2 were highly expressed in 100% (n = 17) of high stage (III-IV) uterine leiomyosarcoma specimens. In addition, TEM1 expression was positively correlated with MMP-2 expression in uterine leiomyosarcoma. The successful establishment of in vitro uterine sarcoma models was confirmed with qPCR and Western blotting tests. TEM1 promoted the invasion and metastasis of uterine sarcoma in vivo and in vitro. MMP-2 expression and activity were up-regulated in hTEM1 cells but down-regulated in shRNA-TEM1 cells. Importantly, MMP-2 knockdown impaired the invasive and migratory capacity of hTEM1 cells. TEM1 promoted ECM remodeling by increasing cell-ECM adhesion and ECM degradation. TEM1 overexpression also induced the formation of invadopodia.

**Conclusion:**

TEM1 was co-expressed and positively correlated with MMP-2 in uterine leiomyosarcoma specimens. In addition, both TEM1 and MMP-2 were associated with tumor development. TEM1 promoted uterine sarcoma progression by regulating MMP-2 activity and ECM remodeling.

**Supplementary Information:**

The online version contains supplementary material available at 10.1007/s12672-023-00613-6.

## Introduction

Uterine sarcomas are rare gynecological malignancies affecting 3–9% of all uterine cancers. Uterine leiomyosarcomas (uLMS) account for 60–70% of all uterine sarcomas compared with 10% for endometrial stromal sarcoma and undifferentiated uterine sarcomas [1]. The prognosis of patients with uterine sarcoma is usually poor because of its aggressiveness and early metastatic nature. A recent US Surveillance reported a 42% overall five-year survival rate for uterine leiomyosarcomas patients and death within 5 years for all patients with late stage (stage III–IV) uterine leiomyosarcomas [2]. Currently, Systemic chemotherapy is the mainstay treatment for patients after enbloc surgery [3].

TEM1 (tumor endothelial marker 1), also known as endosialin or CD248, is dynamically expressed on pericytes, fibroblasts of tumor stroma, but barely expressed in benign and normal tissues [4–6]. Recent studies have found that TEM1 is overexpressed in solid tumors and participate in tumor angiogenesis and tumor progress [7–10]. Ontuxizumab (MORAb-004), a humanized anti-TEM1 monoclonal antibody, was recently studied for its anti-solid tumor efficacy in preclinical and clinical trials [11–14]. Previously, we reported that the TEM1 protein was highly expressed in 19 subtypes of sarcomas, including tumor vessels and tumor cells, with an overall positive rate of 96% [15]. All 13 samples of uterine leiomyosarcoma were TEM1-positive, among which 9 were positive in both tumors and vessels whereas 4 were positive in vessels only. TEM1 is usually up-regulated in tumor cells independent of the TEM1 expression levels in vessels or pericytes [7, 15], indicating that other than angiogenesis, TEM1 might play multiple roles in tumorigenesis and progression of uterine sarcoma.

The extracellular matrix (ECM) provides a dynamic supportive structure for cellular components and modulates tissue homeostasis [16]. the degradation and remodeling of ECM is considered as an essential step in the metastatic dissemination of cancer cells, which facilitates cancer progression [17]. Evidence showed that TEM1 interaction with ECM proteins promoted cell adhesion and migration [6]. In benign and/or malignant tissues, TEM1 is co-expressed with ECM components and enzymes involved in ECM remodeling, including collagens I, III, V, XIII, MMP-2, MMP-14 and lysyl oxidase (LOX) [16, 18]. These ECM-related molecules also participate in angiogenesis and epithelial-mesenchymal transition (EMT). TEM1 protein was reported to bind to fibronectin (FN) and mediate cell adhesion and migration in vivo [6]. An in vivo study showed the possible association between TEM1 and MMP-9 in fibrosarcoma cells [19]. Other studies found that TEM1 and MMP-2 were colocalized in tissue areas of early angiogenic processes [18, 20], suggesting a possible functional association between TEM1 expression and MMPs activity in cell migration and invasion. However, there are limited data supporting the association between TEM1 and MMP-2 and their roles in uterine sarcoma.

In the last decade, studies on TEM1 have mostly focused on its role in promoting angiogenesis [18, 21–23] but not its robust interaction with ECM components in tumorigenesis. The expression of TEM1 and MMP-2 and their correlation with uterine leiomyosarcoma was explored. By establishing TEM1 overexpression and knockdown MES-SA cell models, we demonstrated that TEM1 regulated the expression of MMP-2 and promoted the invasion and migration of uterine sarcoma cells through ECM remodeling.

## Materials and methods

### Clinical specimens

Clinical sarcoma specimens were collected from patients at Tongji Hospital 2015 to 2020, which is affiliated to Tongji University. The study protocol was approved by Ethics Committee of Tongji Hospital. Patients who provided informed consent to undergo staging using FIGO staging system were enrolled [24]. Specimens were obtained during surgeries and stored in liquid nitrogen preserved of paraffin block. Standard formalin-fixed paraffin-embedded (FFPE) techniques were conducted as previously described [15]. Briefly, FFPE tissue sections were blocked in matched normal sera after dewaxing and epitope retrieval. Slides were incubated with primary and second antibodies. Subsequently, the sections were stained with Hematoxylin (Beyotime, China) for 10 min before observation. The slides were observed using the Bond Polymer Refine Detection System. Protein expression levels were independently determined by two senior pathologists on a scale of 0, 1 + , 2 + and 3 + (0 = negative, 1 +  = weak, 2 +  = medium, 3 +  = strong) according to the staining intensity of vasculature, stromal cells and/or tumor cells. The Long H-score was calculated by multiplying overall staining intensity with the percentage of positive cells [25–28]. The staining intensity scores ranged from 0 to 3 and the positive percentage increased from 0 to 100. Theoretically, the final H-score values obtained would range from 0 to 300.

### Cells and reagents

MES-SA cell line was obtained from the National Collection of Authenticated Cell Cultures (Shanghai, China) and cultured in McCoy’s 5α medium (Invitrogen, USA) supplemented with 10% FBS (Invitrogen, USA) and 100 Unit/ml penicillin/streptomycin (Sangon Biotech, China). Cells were cultured at 37 °C and in 5% CO_2_ incubator following the American Type Culture Collection (ATCC) guidelines. Anti-TEM1 (ab67273), anti-MMP-2 (ab92536) and GAPDH (ab9485) antibodies were purchased from Abcam (Cambridge, MA, USA).

### Establishment of TEM1 overexpression and knockdown cell lines

A customized TEM1-overexpression lentivirus vector was constructed by KeyGen Biotech (Nanjing, China). MES-SA cells were transfected with the TEM1-lentivirus (LV-hTEM1-6His-OE) or control lentivirus (LV-vector) and selected using 2 µg/ml puromycin (Sigma–Aldrich, St. Louis, MO, USA). Selected cells were cultured in conditioned medium without puromycin to generate multiple monoclonal cell lines. The cells were subjected to Western blot analysis to detect TEM1 expression.

Short hairpin RNAs (shRNAs) targeting TEM1 (shRNA-TEM1) were used to knock down TEM1. Commercially inventoried lentivirus vectors expressing shRNA (LV-Htem1-shRNA) and negative control (LV-NC-shRNA) were constructed by KeyGen Biotech (Nanjing, China) with the following sequences: shRNA-TEM1 sequence: (sense) 5’-CACCGGTGGCTTCGAGTGTTATTGTCTCGAGACAATAACACTCGAAGCCACCTTTTTTC-3’, (antisense) 5’-AAAAGGTGGCTTCGAGTGTTATTGTCTCGAGACAATAACACTCGAAGCCACCAAAAAAGAGCT-3’; shRNA-NC (scramble) sequence: (sense) 5′‐TAACTAGTAACGGCTGCTCCCTCGAGGGAGCAGCCGTTACTAGTTTTTTTTC‐3′; antisense: 5′‐ATTGATCATTGCCGACGAGGCTCGAGCCTCGTCGGCAATGATCAAAAAAAAGAGCT‐3′. And pLL3.7 vector containing shRNA-TEM1 or shRNA-NA was combined with pRSVRev vectors and pMD.G vectors (Clonetech, USA). Subsequently, packaging vectors and lentiviral vector DNAs and were transfected into 293 T cells and harvested after 72 h. Supernatants containing lentiviruses were purified, concentrated and quantified. MES-SA cells were transfected with LV-hTEM1-shRNA or LV-NC-shRNA lentivirus and selected using 2 µg/mL puromycin.

### RNA extraction, cDNA synthesis and real-time quantitative PCR

Total RNA was extracted using TRIzol reagent and reverse transcribed using High-capacity cDNA Reverse Transcription Kit. The cDNA samples generated were subjected to qPCR analysis using TaqMan Universal Master Mix II and commercially inventoried primers for TEM1 and MMP-2 (Applied Biosystems, USA). cDNA samples were then quantified using an ABI ViiA 7 System (Applied Biosystems, USA). 18S probe was used as an endogenous control. The reaction program was set as follows: 95 °C for 15 min followed by denaturation (95 °C for 10 s), annealing (for 30 s), and elongation (for 30 s). In total, 40 cycles of annealing and elongation steps were performed. The qPCR reactions were repeated three times and 2^−ΔΔCT^ method was used to quantify relative gene expression levels.

### Western blot

Total protein was extracted from cell lysates or tumor specimens. Protein concentrations were determined using a bicinchoninic acid (BCA) kit (Sangon Biotech, China). Total protein (20 μg per sample) was electrophoretically separated on a 4–20% gradient sodium dodecyl sulfate polyacrylamide gel (SDS-PAGE) and transferred to PVDF membranes (Invitrogen, USA). The blots were incubated with 5% non-fat milk and probed with TEM1 (1:5000 dilution) and MMP-2 (1:2500 dilution) antibodies. The secondary antibody horseradish peroxidase (HRP) conjugated with goat anti-rabbit immunoglobulin G (1:5000 dilution) was then added to the membranes before protein detection. An endogenous glyceraldehyde 3-phosphate dehydrogenase (GAPDH) antibody (1:5000 dilution) was used as a control. Reactive proteins were detected using TMB Substrate Solution (Beyotime, China) through GBOX chemiXR5 imaging system (Gene company, Hongkong, China).

### siRNA transfection and knockdown

MMP-2 siRNA was constructed using previously published sequences [29] by GenePharma (Shanghai, China). MES-SA cells were grown in serum-free medium to 50% confluency. Cells were then transfected with 20 μmol/L MMP-2 siRNA or Scramble siRNA using lipofectamine 2000 and incubated for 6 h. Cells were washed and cultured in the conditioned medium for observation at 24, 48 and 72 h. The expression of MMP-2 protein was detected using Western blot. MMP-2 siRNA sequence: F) 5-AACACAGCCTTCTCCTCCTG, R) 5-CACCTACACCAAGAACTTCC; β-actin sequence: F) TTGCGTTACACCCTTTCTTG, R) ACTGCTGTCACCTTCACCG.

### Gelatin zymography

The cell supernatant was concentrated and mixed with 4X non-reducing sample buffer (Invitrogen, USA). Equivalent amounts of total protein supernatants (8 µg each lane) were loaded on a 7.5% SDS-PAGE gel containing 1 mg/mL gelatin. After separation, the gels were washed for 30 min in 2.5% Triton X-100 (Invitrogen, USA) to remove the SDS and incubated in developing buffer (Invitrogen, USA) for 15 h at 37 °C. The gels were then rinsed with distilled water and stained with Coomassie blue to detect MMP-2 activity.

### Cell migration assay

The migration ability of cells was determined using the transwell migration assay (Corning, New York, USA). Briefly, serum-free medium was added to the upper chamber containing 1 × 10^5^ cells and 20% serum-contained medium was added in the lower chamber. After incubation overnight, cells on the upper membrane of upper chamber were removed clearly, and migrated cells on the lower membrane were fixed with 4% paraformaldehyde, stained with crystal violet and counted under optical microscope. The percentage of migrated cells was calculated as the ratio of migratory cells against the total number of seeded cells. All assays were performed three times.

### Cell invasion assay

The cell invasion ability of cells was evaluated using the transwell invasion assay. Briefly, the upper chamber was precoated with 0.5 mg/ml Matrigel matrix and serum-starved MES-SA cells (1 × 10^5^) were added. In addition, medium containing 20% serum was added to the lower chamber. The chambers were incubated 72 h, after which cells that invaded to the lower membrane of upper chamber were fixed with 4% paraformaldehyde and stained with crystal violet. The number of invaded cells was immediately counted under a microscope. All assays were performed three times.

### ECM adhesion assay

96-well plates were precoated with human fibronectin, laminin, or vitronectin overnight at 4 °C and blocked with 1% BSA for 1 h at 37 °C. Cells were added to the plates at 1 × 10^4^ cells per well and incubated at 37 °C for 2 h. The wells were washed twice with PBS. Next, 200 µL serum-free medium was added to the wells and the OD value was tested using the CCK-8 assay kit.

### ECM degradation assay

The gelatin degradation assay was conducted as described by Artym et al. [30]. Briefly, 18 mm acid washed coverslips were seeded in 12-wells tissue-culture plate. 1 mL of 50 μg/mL solution of poly- L-lysine was added to each well and incubated for 20 min at room temperature. The coverslips were washed three times with PBS and then incubated with 1 mL of 0.5% glutaraldehyde for 15 min at room temperature. The coverslips were washed again with PBS and coated with 0.2 mg/ml FITC-coupled gelatin (G-13187, ThermoFisher, USA) for 15 min. 3 × 10^4^ of MES-SA or MBA-MD-231 cells were seeded on gelatin-coated coverslips for 5 to 8 h and then immunofluorescence was measured. Ten random fields per sample were captured using a laser confocal microscope to calculate gelatin degradation. To better observe the degradation behavior of cells, three-dimensional (3D) videos were reconstructed by collecting sequential Z-sections. Image J software was used to distinguish degraded (black) areas from nondegraded (green) area by thresholding. The percentage of degradation for each field was calculated based on the ratio of degraded regions to total regions.

### In vivo study

Fifteen BALB/C nude female mice (4 weeks old; 10–12 g; Viton Lever, Beijing, China) were divided into three groups (five mice per group): the hTEM1-MES-SA cell group, shRNA-TEM1-MES-SA cell group and NC-MES-SA cell group. The cell density in each group was adjusted to 5 × 10^6^ cells/mL and 0.2 mL was injected subcutaneously into the left armpit of the nude mice. Mice were housed in a semi-barrier system with constant temperature and humidity. The animals were euthanized after 4 weeks. The tumor volumes were measured and calculated using the following formula: volume (cm^3^) = (width^2^ × length)/2.

Another fifteen BALB/C nude female mice were randomly divided into three groups with five mice in each group. 1 × 10^6^ of MES-SA shRNA-TEM1 cells, hTEM1 cells and NC cells in 0.2 ml of PBS was injected intraperitoneally. The mice were sacrificed on day 28 post transplantation and the number of metastases were calculated.

All animal experiments were performed in accordance with the institutional guidelines and approved by the Institutional Animal Care and Use Committee of Tongji Hospital of Tongji University.

### Statistical analysis and data presentation

Pearson’s correlation and linear regression analyses were conducted to determine the correlation between TEM1, MMP-2 and MMP-9 expression in clinical specimens. For qPCR tests, 18 s probe was used as the internal control. In the Western blotting test, protein expression was normalized to the expression of GAPDH. Statistical and graphing data were analyzed using GraphPad Prism 5 software. Graphical data were presented as the mean values with standard deviations. Comparisons between groups were analyzed using unpaired student's *t*-test or Two-way ANOVA. A *P* value of less than 0.05 was considered statistically significant. Each experiment was repeated at least two times in  triplicates.

## Results

### TEM1 and MMP-2 are co-expressed in uterine leiomyosarcoma but not in leiomyoma specimens

Our previous study found that TEM1 was overexpressed in 19 sarcoma subtypes [15]. Here, we studied TEM1, MMP-2 and MMP-9 expression in 50 samples of uterine leiomyosarcoma (n = 25) and leiomyoma (n = 25) tissues (Table 1). The expression levels of TEM1, MMP-2 and MMP-9 were quantified using Long H-scoring system, as previously described [31–33]. Our analysis revealed that TEM1 and MMP-2 were expressed in 92% (23 positive) and 88% (22 positive) of the 25 uterine leiomyosarcoma tissues, respectively. In contrast, only weak (median of 20 and 35 for TEM1 and MMP-2, respectively) or negative TEM1 (10 negative) and MMP-2 (18 negative) staining were seen in 25 leiomyoma tissues. According to FIGO classification, 8 specimens were low-grade (stage I-II) and 17 were high-grade (stage III-IV). TEM1 and MMP-2 were highly expressed in all 17 of high stage (III-IV) uterine sarcoma specimens and 62.5% or 5 of 8 of low-grade specimens. Interestingly, previous studies demonstrated a possible association between TEM1 and MMP-9 in fibrosarcoma cells [19]. However, in our study, MMP-9 was 72% negative (21 of 25) or only weakly expressed (median Long-H score of 80) in uterine leiomyosarcoma which was similar to that of leiomyoma tissues (22 of 25 negative). Only weak or negative staining of TEM1 (9 positive, 45%), MMP-2 (3 positive, 15%) and MMP-9 (2 positive, 10%) were detected in normal uterine myometrium. This suggested that MMP-9 might not play an important role in tumorigenesis of uterine leiomyosarcoma (Table 1).

**Table 1:** TEM1, MMP-2 and MMP-9 expression in，uterine leiomyosarcoma (uLMS), leiomyoma and normal uterine tissues

Group	Total (n)	FIGO classification	N (n)	TEM1 expression^A^					MMP-2 expression^B^					Mmp-9 expression^C^				
				Neg	Min	Max	Mean	Total positive	Neg	Min	Max	Mean	Total positive	Neg	Min	Max	Mean	Total positive
uLMS	25	I	4	2	0	144	43.5	23 (92%)	2	0	95	28.8	22 (88%)	4	0	0	0	4 (16%)
		II	4	0	80	235	146.3		1	0	170	77.5		4	0	0	0	
		III	8	0	80	230	162.9		0	35	180	99.1		5	0	85	20	
		IV	9	0	35	300	236.1		0	10	190	135.6		8	0	160	17.8	
Leiomyoma	25	/	/	10	0	120	20	15 (60%)	18	0	85	35	7 (28%)	22	0	65	15	3 (12%)
Normal tissues	20	/	/	11	0	70	12.5	9 (45%)	17	0	30	3.0	3 (15%)	18	0	20	2.0	2 (10%)

^A^The mean of TEM1 Long H-Score are positively correlated with tumor stages, r = 0.9554, *p* < 0.01

^B^The mean of MMP-2 Long H-Score are positively correlated with tumor stages, r = 0.9602, *p* < 0.01

^C^The mean of MMP-9 Long H-Score are not correlated with tumor stages, r = 0.1205, *p* > 0.05

**Fig. 1:**
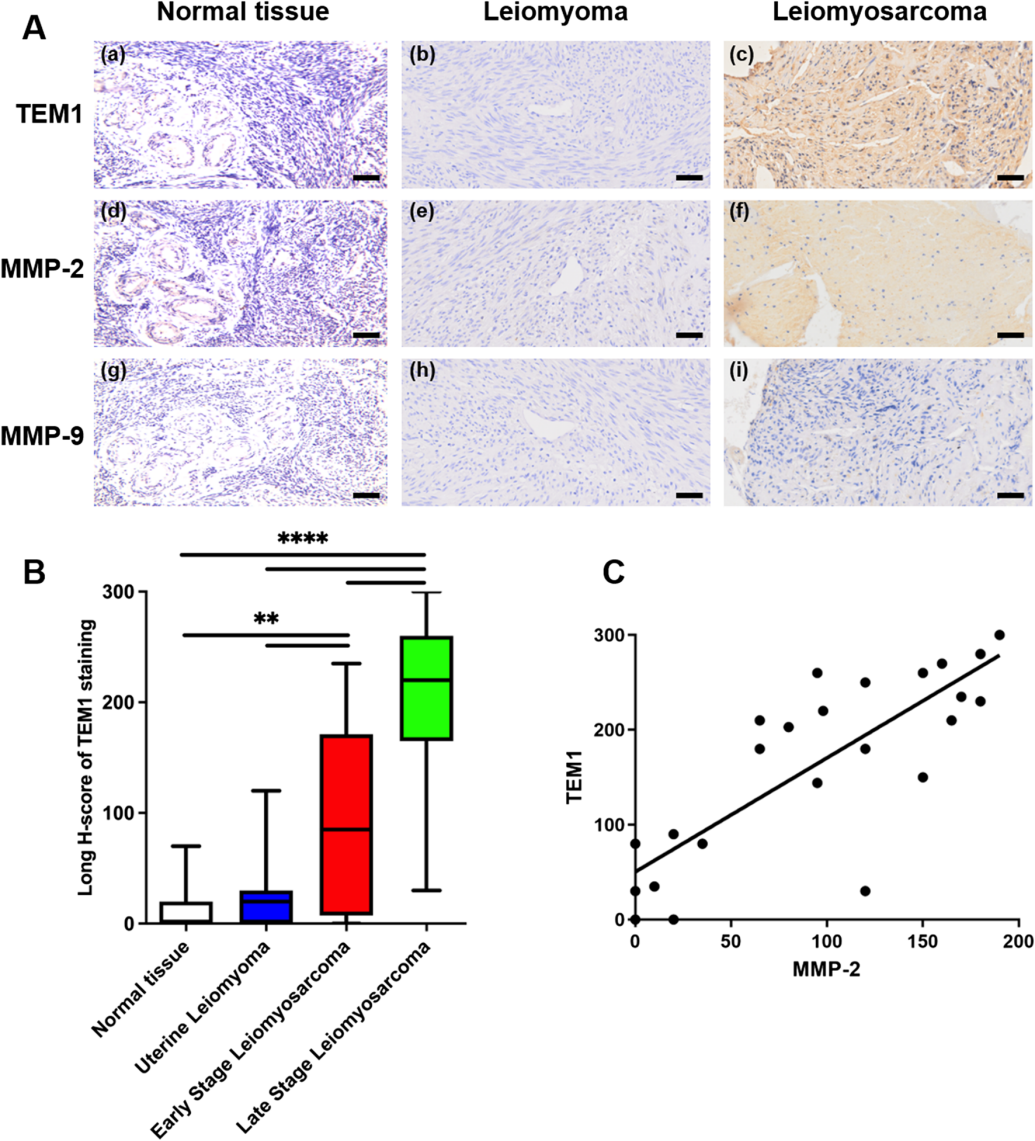
The expression of TEM1, MMP-2 and MMP-9 in different uterine samples. **A** Representative immunohistochemical results of TEM1, MMP-2 and MMP-9 staining. TEM1 and MMP-2 were negative in uterine myometrium (normal tissue) and leiomyoma, strong positive in leiomyosarcoma; MMP-9 staining was negative or weakly expressed in all samples. Scale bar, 50 μm. **B** Boxplot for Long H-scores of TEM1 in normal myometrium, leiomyoma and leiomyosarcoma tissues. TEM1 Long H-scores were positively correlated with tumor stages, r = 0.9554. **, *P* < 0.01; ****, *P* < 0.0001. **C** TEM1 expression was positively correlated with MMP-2 expression in uterine leiomyosarcoma tissues. Correlation curves are shown with r square, and *P*‐values from Pearson´s correlation analysis. R square = 0.6487, Pearson r = 0.8054, *P* < 0.0001

Figure 1 A shows representative images of TEM1 (a–c), MMP-2 (d–f) and MMP-9 (g–i) stained tissues from a patient with uterine myometrium (normal tissues), a patient with leiomyoma and another patient with stage III uterine leiomyosarcoma using serial tumor sections. The Long H-scores of TEM1, MMP-2 and MMP-9 were 280 (80% 3 + , 20% 2 +), 130 (70% 1 + , 30% 2 +) and 0 in this stage III leiomyosarcoma tissue. The boxplot showed that TEM1 staining was positive in 92% of leiomyosarcoma tissues with a median score of 203 (Range, 0–300), significantly higher than those of leiomyoma tissues (*P* < 0.01; Fig. 1B) and uterine myometrium tissues (*P* < 0.0001; Fig. 1B). What is more, the Long H-scores of TEM1 in late stage uterine leiomyosarcoma was significantly higher than those with early stage (*P* < 0.01; Fig. 1C)

Altogether, these results indicate that TEM1 and MMP-2 were co-expressed in uterine leiomyosarcoma tissues, especially high-grade lesions, suggesting poor prognosis in uterine leiomyosarcoma patients. MMP-2, but not MMP-9, might work together with TEM1 in uterine leiomyosarcoma progression.

### TEM1 and MMP-2 expression in uterine leiomyosarcoma tissues are positively correlated

To determine the correlations among TEM1, MMP-2 and MMP-9 in uterine leiomyosarcoma tissues, protein expression levels were calculated using Long H-scores and Pearson’s correlation across all patients. Table 1 shows that the median values of both TEM1 and MMP-2 Long H-Score were positively correlated with tumor stages (TEM1, Pearson’s r = 0.9554, *P* < 0.01; MMP-2, Pearson’s r = 0.9602, *P* < 0.01). However, we found no correlation between median MMP-9 Long H-Score values and tumor stages (Pearson’s r = 0.1205, *P* > 0.05). Moreover, the correlation curve in Fig. 1C revealed a significantly positive correlation between TEM1 and MMP-2 expression (r squared = 0.6487, Pearson r = 0.8054, *P* < 0.0001), suggesting a functional association between TEM1 and MMP-2 in promoting tumor progression of uterine leiomyosarcomas. However, no correlation was found between TEM1 and MMP-9 expression (r square = 0.0202, Pearson r = 0.1420, *P* = 0.5999).

### TEM1 overexpression and knockdown in vitro model are established

**Fig. 2:**
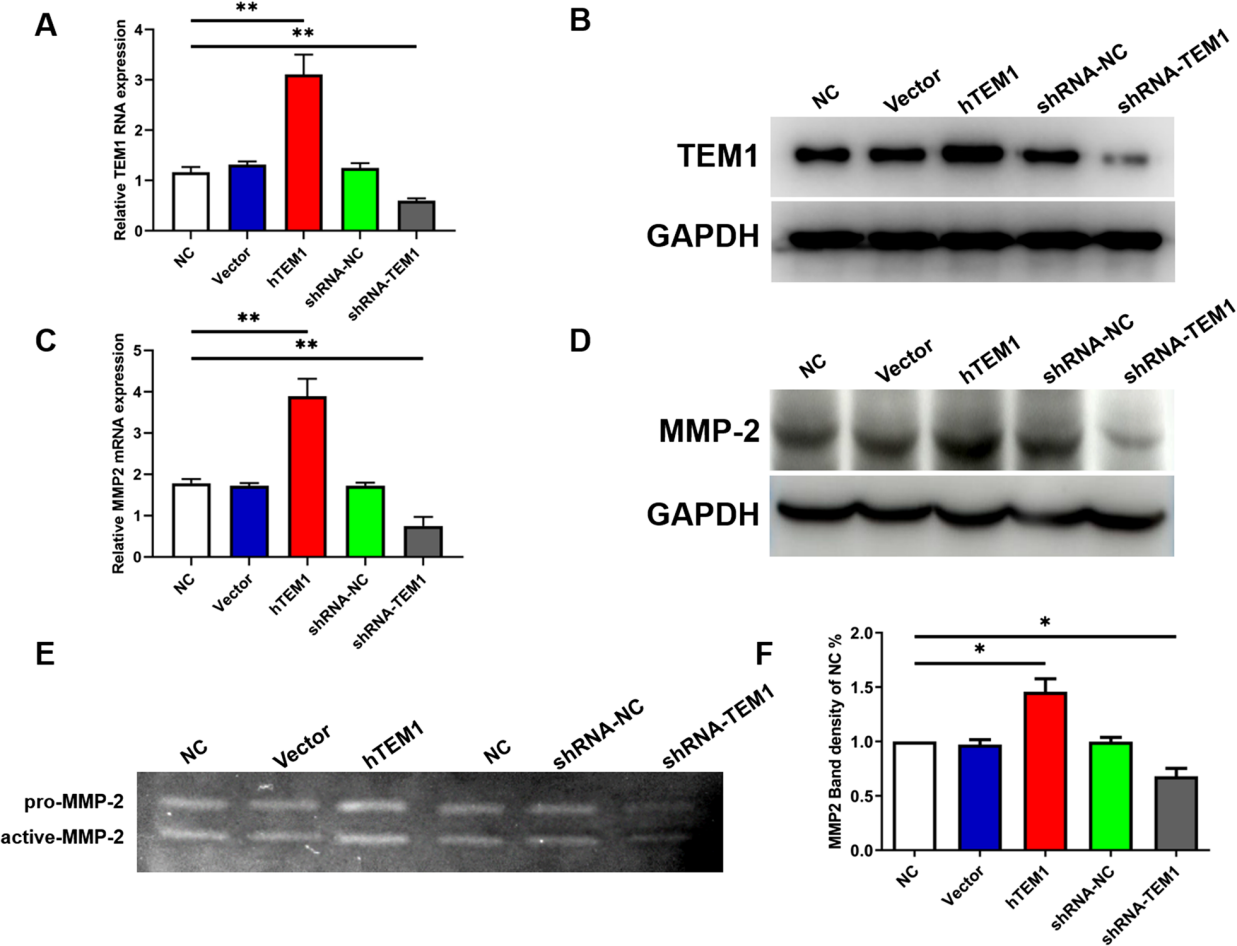
The expression level of MMP-2 regulated by TEM1. Lentivirus-mediated hTEM1 MES-SA control vector (Vector), TEM1 overexpression vector (hTEM1), shRNA NC vector (shRNA-NC) and shRNA-TEM1 vector were transfected into MES-SA cell lines (NC) for the establishment of TEM1 overexpression and low expression MES-SA cell lines. TEM1 mRNA **A** and protein **B** expression were determined using qPCR and Western blot assays, respectively. The protein expression level of MMP-2 mRNA **C** and protein **D** in all cell lines. MMP-2 expression was upregulated in hTEM1 cells and downregulated in shRNA-TEM1 cells. Gelatin zymography showing elevated expression of MMP-2 in hTEM1 cells and decreased expression of MMP-2 in shRNA-TEM1 cells **E**, based on band density using Image J (F). *, *P* < 0.05; **, *P* < 0.01

TEM1 overexpression (hTEM1) and knockdown (shRNA-TEM1) MES-SA cell lines were established using a lentivirus-mediated TEM1 vector and shRNA. Compared with control vector MES-SA (vector) cells, TEM1 mRNA and protein levels were significantly higher in hTEM1 MES-SA cells using qPCR and Western blot (Fig. 2A, B). In contrast, TEM1 mRNA and protein expression levels were significantly lower in shRNA-TEM1 MES-SA cells (shRNA-TEM1) than in shRNA-NC-TEM1 MES-SA (shRNA-NC) cells (*P* < 0.01). TEM1 mRNA and protein expression levels in vector and shRNA-NC group were not significantly different from those of non-treated negative control wild-type MES-SA (NC) cells (*P* > 0.05).

### TEM1 regulates expression of MMP-2 in MES-SA cells

We determined MMP-2 mRNA and protein expression levels in hTEM1 MES-SA (hTEM1) cells, shRNA-TEM1 MES-SA (shRNA-TEM1) cells, shRNA-NC-TEM1 MES-SA (shRNA-NC) cell, vector MES-SA (vector) cells and wild-type MES-SA (NC) cells using qPCR and Western blot (Fig. 2C, D). Results showed that MMP-2 mRNA and protein expression levels were significantly higher in hTEM1 cells than in vector cells (*P* < 0.01). In addition, MMP-2 mRNA and protein expression levels were significantly down-regulated in shRNA-TEM1 cells, compared with shRNA-NC cells (*P* < 0.01). To determine whether the activation of MMP-2 was also up-regulated by TEM1, we conducted gelatin zymography with cell supernatants from each group (Fig. 2E). Compared with vector cells, hTEM1 cells exhibited a higher level of activated MMP-2 with a higher band density, whereas shRNA-TEM1 cells showed a lower level of activated MMP-2 with a lower band density (both *P* < 0.05; Fig. 2F). These results suggested that TEM1 up-regulated MMP-2 expression and activity in MES-SA cells.

### Loss of MMP-2 inhibits migration and invasion ability of hTEM1 MES-SA cells

**Fig. 3:**
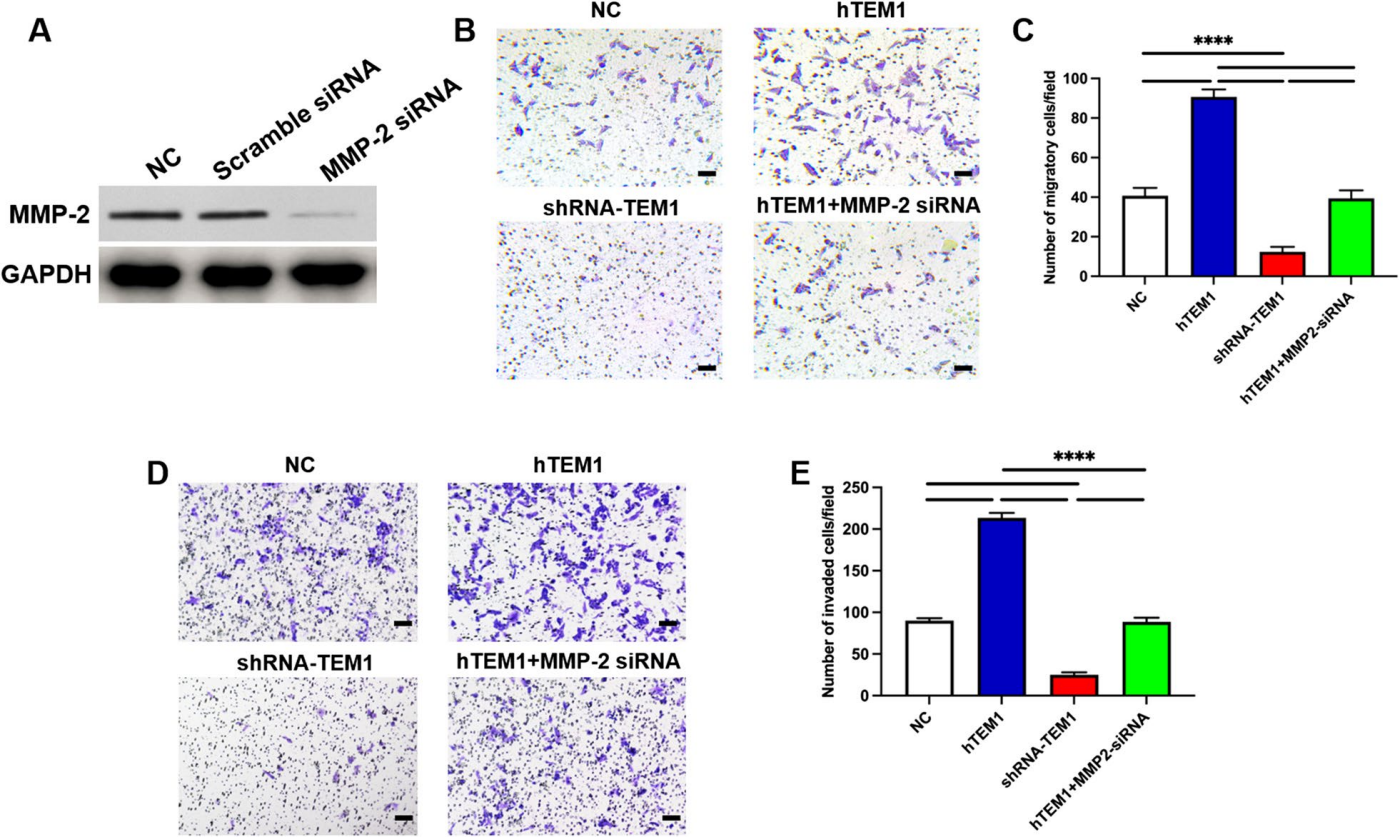
TEM1 regulating MES-SA cell migration and invasion via MMP-2. Western blotting results confirming knock-down of MMP-2 protein by MMP-2 siRNA **A**. Both cell migration ability **B** and **C** and invasion ability **D** and **E** were enhanced in hTEM1 cells and inhibited in shRNA-TEM1 treated cells. And hTEM1 MES-SA cells transfected with MMP-2 siRNA (hTEM1 + MMP-2 siRNA) showed comparable invasion and migration ability to NC group. ****, *P* < 0.0001. All assays were replicated three times in triplicates. Magnification, 100X; bar scale, 100 μm

Our limited data [34] suggested that TEM1 promoted migration and invasion of MES-SA cells. However, it is unknown whether MMP-2 participates in TEM1-promoted migration and invasion of MES-SA cells. Effect of *MMP-2* gene knockdown in hTEM1 MES-SA cells using an MMP-2-targeted siRNA were confirmed by Western Blot (Fig. 3A). The invasion and migration abilities of cells were subsequently determined using transwell assays. The migration and invasion abilities of hTEM1 MES-SA cells were inhibited by MMP-2 siRNA (both *P* < 0.05) and were not significantly different from those of control cells (*P* > 0.05; Fig. 3B–D).

### MMP-2 is necessary for TEM1- mediated ECM adhesion

**Fig. 4:**
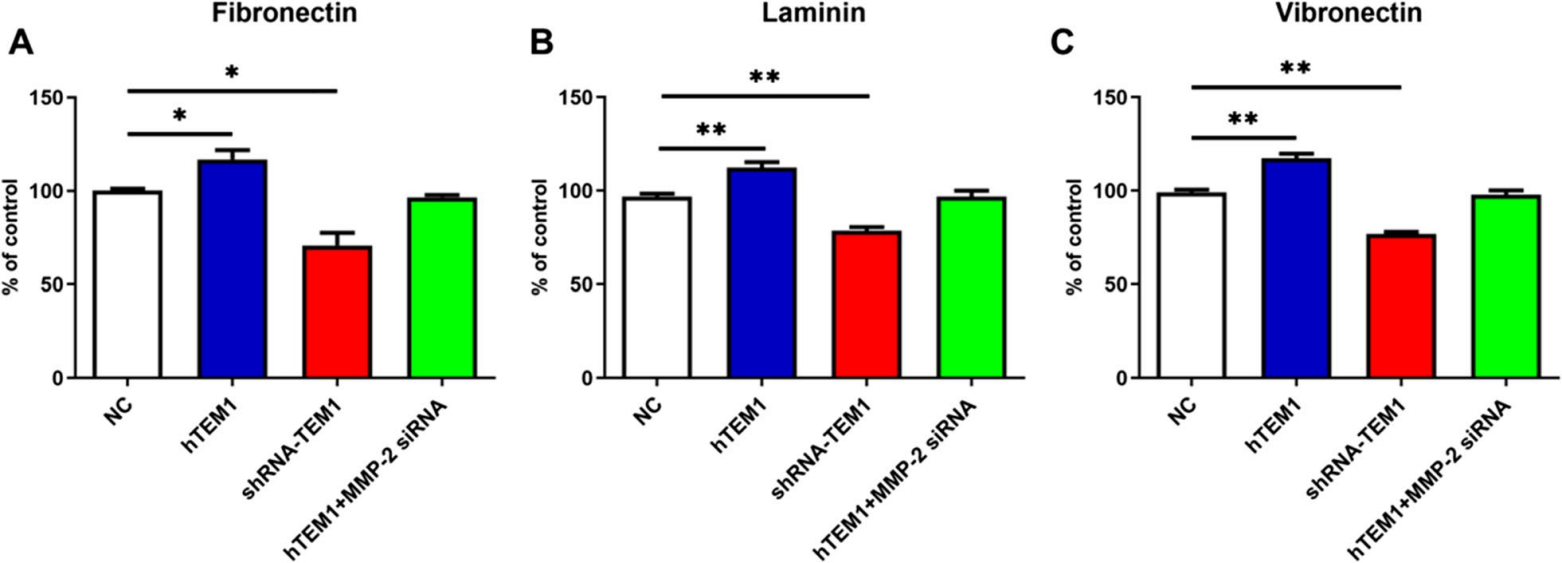
TEM1 promoted MES-SA cell adhesion to extracellular matrix (ECM) via MMP-2. NC, hTEM1, shRNA-TEM1, and hTEM1 + MMP-2 siRNA cells were selected to adhere to fibronectin (Fn) **A**, laminin (Laminin) **B**, and Vitronectin (Vn) **C** in the ECM-coated 12-well plate for 72 h respectively. The numbers of adhesion cells were calculated based on the CCK-8 assay. *, *P* < 0.05; **, *P* < 0.01. All assays were replicated three times in triplicates

Cell-ECM adhesion is a critical step in cancer distant metastasis [35–37]. Previous studies revealed that TEM1 co-expressed with ECM components and promoted the adhesion of cells to fibronectin, increasing cell migration in vitro [18]. Our study explored changes in TEM1-mediated adhesion ability of MES-SA cells to ECM components, including fibronectin, laminin and vitronectin, using Cell-ECM adhesion assay. As shown in Fig. 4A–C, hTEM1 cells displayed higher adhesion ability to ECM components compared with negative control cells (all *P* < 0.05). MMP-2 siRNA significantly decreased the number of adhered hTEM1 cells in each group (*P* < 0.5), leading to its adherent ability not significantly different from negative control cells (*P* > 0.05). These results indicated that TEM1 promoted the adhesion ability of MES-SA cells to ECM components in the presence of MMP-2.

### MMP-2 is necessary for TEM1-mediated ECM degradation

**Fig. 5:**
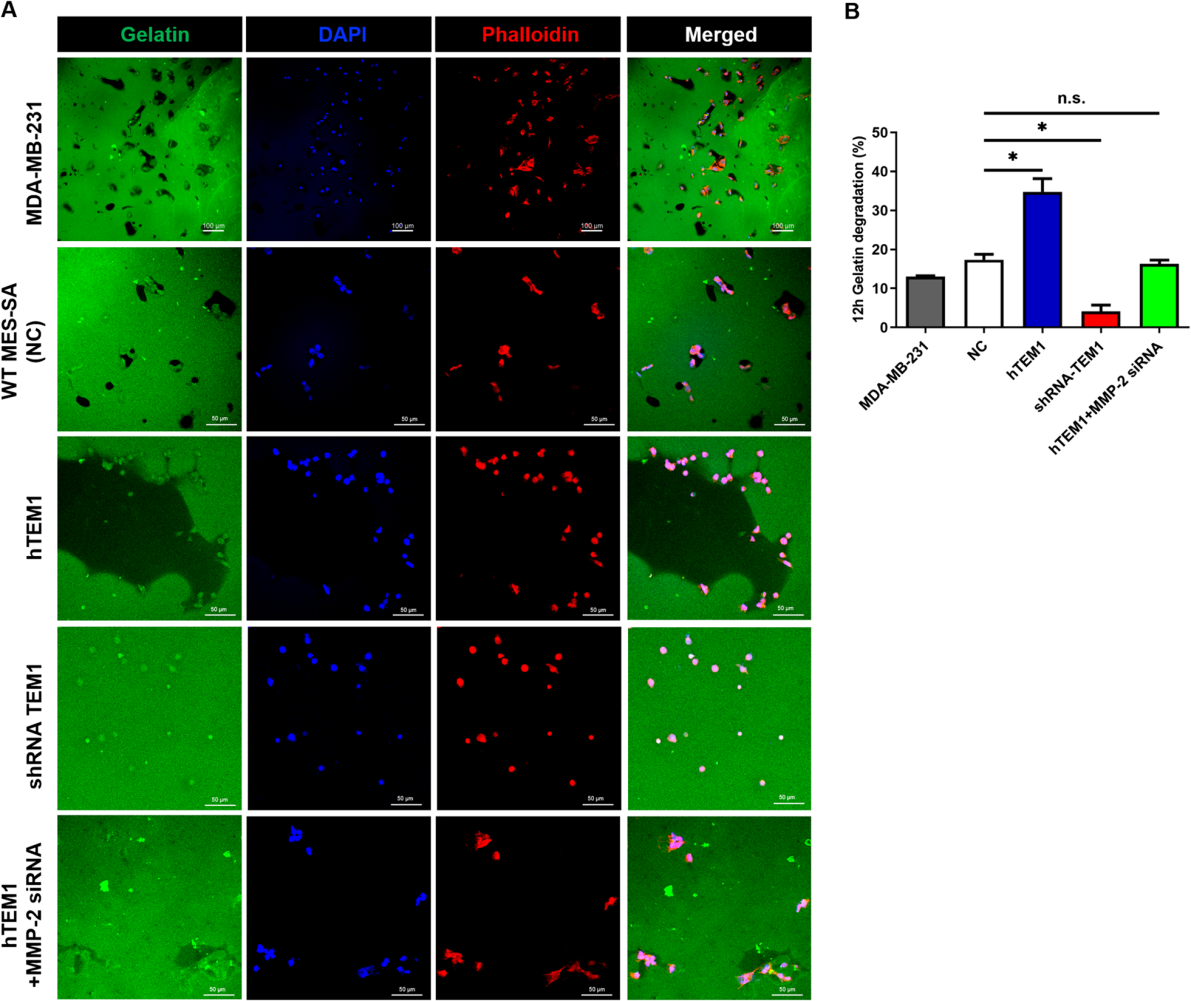
(**A**) Gelatin degradation assays of MDA-MB-231, WT MES-SA (NC), hTEM1 MES-SA, shTEM1 MES-SA and hTEM1 MES-SA knocked down MMP-2 expression by MMP-2 siRNA (hTEM1 + MMP-2 siRNA) after 12 h. MDA-MB-231 served as positive control. Pool-like areas of degradation in gelatin matrix formed around hTEM-MES-SA cells. Immunofluorescence staining of FITC- conjugated gelatin (green), Phalloidin (red), nuclei (blue), and merged images. (**B**) Quantification of gelatin degraded area (n = 10) by Image J. Data are presented as mean ± standard deviation (SD). *, *P* < 0.05 in Student *t*-test; n.s., none-sense, *P* > 0.05; Scale bar, 100 μm or 50 μm

To study the ability of MES-SA cells to degrade the matrix, cells were cultured on coverslips coated with FITC-conjugated gelatin matrix for 12 h and evaluated with confocal microscopy (Fig. 5A). Breast cancer cell line MDA-MB-231 was included as a positive control (PC) because its stable capability to degrade ECM has been confirmed in literature [30, 38–40]. After 12 h of incubation, black areas of degradation were shown underneath the cell body, and cells were found within the degraded areas. Similar degradation pattern was seen in WT MES-SA (NC) cells as black holes were observed in gelatin matrix where the cells were located. Interestingly, in hTEM1 MES-SA cells, we observed pool-like areas of degradation in gelatin matrix, while when *TEM1* was knocked-down (shRNA-TEM1 group), gelatin degradation decreased significantly compared with WT MES-SA and hTEM1 MES-SA cells. To clarify the role of MMP-2 in TEM1-mediated ECM degradation, we knocked down MMP-2 expression using MMP-2 siRNA in hTEM1 MES-SA cells and found that gelatin degradation was decreased compared with hTEM1 MES-SA group. To quantify the degraded area in different cells, we calculated the percentage of degradation in each field as a ratio of degraded regions to the total region using Image J software. According to the results showed in Fig. 5B, hTEM1 MES-SA cells displayed the highest percentage of degradation (*P* < 0.05, Fig. 5B) followed by MDA-MB-231 cells. While shRNA-TEM1 MES-SA cells had the lowest degradation compared with MES-SA NC cells (*P* < 0.05, Fig. 5B). No significant difference was observed between NC MES-SA cells and hTEM1 + MMP-2 siRNA cells (*P* > 0.05, Fig. 5B).

Notably, in our degradation assays, sometimes we only observed shadows or lower intensity compared with the rest of the green gelatin in gelatin matrix instead of black holes, which is consistent with previous studies [39, 41, 42]. Mazurkiewicz E et al. [41] pointed out that this phenomenon could be an incomplete degradation of gelatin in the three dimension, leading to degraded areas not shown as black hole but only seem to be less intense than the rest of the green FITC-gelatin layer. What is more, when aggressive degradation was seen in some certain cell types including MES-SA, pool-like clear area might be seen in gelatin matrix [40, 43]. This indicates that sometimes two-dimensional images might not efficiently reveal cell behaviors in gelatin matrix as it is impossible to determine whether there are cells inside the pool on a different gelatin layer. In order to better elucidate TEM1-mediated ECM degradation, we reconstructed three-dimensional images by collecting sequential Z-sections based on each field of TEM1-hTEM1 cells and positive control MDA-MB-231 cells (Supplementary video 1 and 2). Compared with two-dimensional (2D) image of a pool-like black degradation area with cells close to the matrix boundaries, the three-dimensional (3D) video (Supplementary video 2) depicted the depth of degraded gelatin inside a pool, with cells presented on different gelatin layers, confirming that hTEM1 cells degraded ECM not only horizontally but also vertically. Moreover, the three-dimensional image clearly outlined the track of aggressive cell migration on different layers of gelatin matrix. We also captured three-dimensional images of gelatin degradation by MES-SA cells, which clearly outlined the cells inside of degraded areas and different layers of degradation (Supplementary images).

**Fig. 6:**
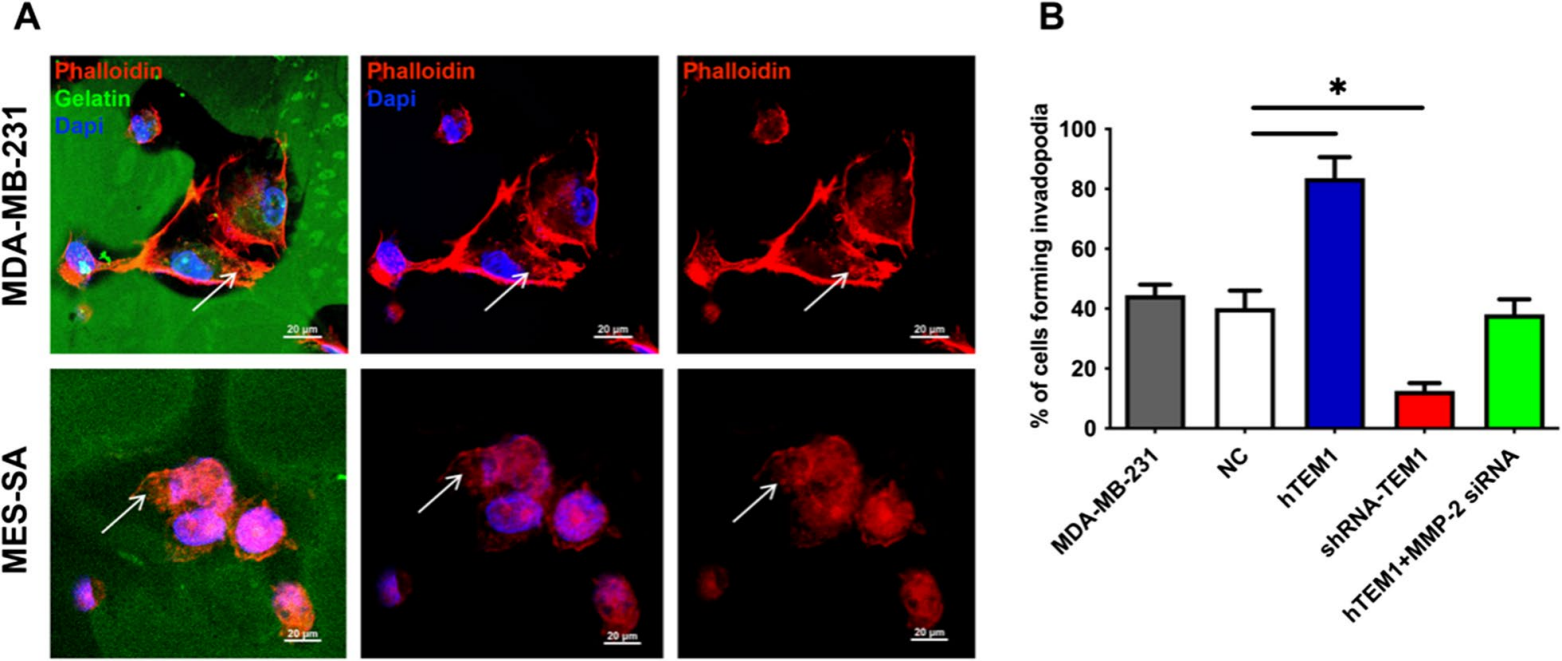
Immunofluorescence of invadopodia. **A** The merged images of F-actin (red), and nuclei (blue) are shown. Arrows indicate invadopodia locations. **B** Quantification of invadopodia in MDA-MB-231 and MES-SA cells. Data are presented as mean ± standard deviation (SD). *, *P* < 0.05. Scale bar, 20 μm

To find out whether TEM1 promoted invadopodia formation ability, representative images of MDA-MB-231 and hTEM1 MES-SA cells were shown in Fig. 6A. F-actin-enriched invadopodia (big rosettes) were observed at the cell edge in both cells (Fig. 6A, white arrows). To quantify invadopodia formation, cells forming more than five F-actin positive invadopodia were counted and normalized to the total cell number of each field. The results (Fig. 6B) showed that compared with WT MES-SA (NC) group, the highest percentage of invadopodia was formed by hTEM1 MES-SA cells (*P* < 0.05, Fig. 6B) and the lowest proportion was found in shRNA-TEM1 cells (*P* < 0.05, Fig. 6B). When *MMP-2* was knocked-down, the invadopodia formation ability of hTEM1 MES-SA cells was impaired. There was no significant difference in invapodia formation ability between hTEM1 + MMP-2 siRNA cells and NC cells (*P* > 0.05, Fig. 6B). These results demonstrated that invadopodia formation was involved in TEM1-mediated degradation and could be inhibited by MMP-2 knockdown.

### TEM1 overexpression promotes the metastasis of uterine sarcoma in the animal model

**Fig. 7:**
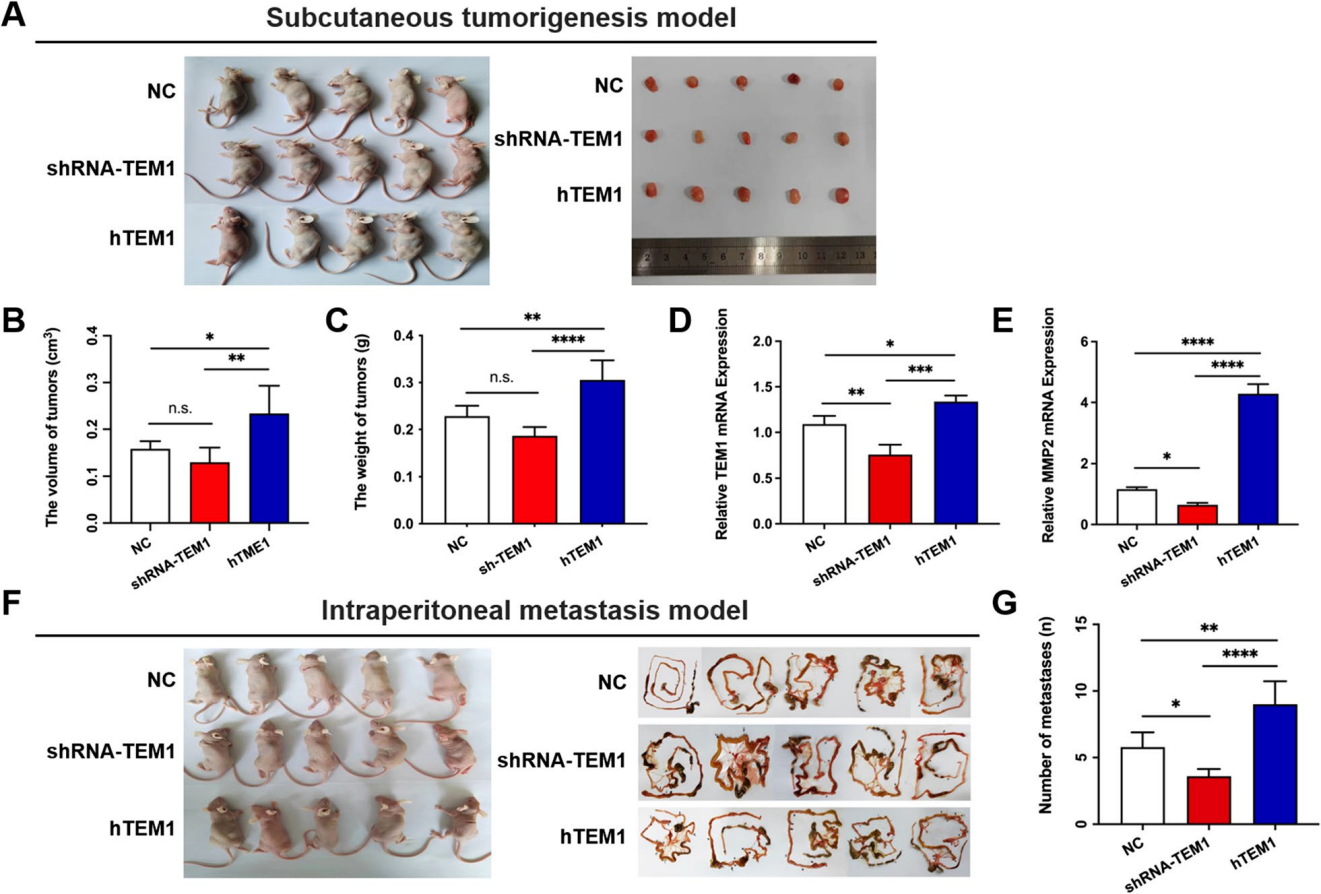
TEM1 promotes the metastasis of uterine sarcoma cells rather than tumor growth. **A** MES-SA NC, shTEM1 and hTEM1 cells were injected subcutaneously in three groups of mice respectively (n = 5 in each group). Tumor weight **B** and volume **C** were measured and calculated. TEM1 mRNA **D** and MMP-2 mRNA **E** expression levels of subcutaneous tumors from each group were determined by RT-qPCR. **F** MES-SA NC, shRNA-TEM1 and hTEM1 cells were injected intraperitoneally in three groups of mice respectively (n = 5 in each group). Metastasis sites were displayed in black arrows and the number of metastases were calculated **G**. n.s., none-sense; *, *P* < 0.05; **, *P* < 0.01; ***, *P* < 0.001; ****, *P* < 0.0001

To validate the role of TEM1 in uterine sarcoma proliferation and metastasis, we conducted in vivo study using BALB/C nude female mice. MES-SA NC, shRNA-TEM1 and hTEM1 cells were injected subcutaneously in three groups of mice respectively (n = 5 in each group) (Fig. 7A). Although the volume and weight of tumors significantly increased in hTEM1 group compared to group (Fig. 7B and C, both p < 0.05), the tumor size and weight were not significantly affected in shRNA-TEM1 group (Fig. 7B and C, p > 0.05). RT-qPCR analysis was used to confirm the overexpression of TEM1 mRNA levels in tumors of hTEM1 group and deletion effect of TEM1 mRNA in those of shRNA-TEM1 group (Fig. 7D). MMP-2 mRNA expression was up-regulated in tumors of hTEM1 group (Fig. 7E, p  < 0.0001) and down-regulated in tumors of shRNA-TEM1 group (Fig. 7E, p  < 0.05), confirming the TEM1 regulation effect on MMP-2.

Furthermore, MES-SA NC, shRNA-TEM1 and hTEM1 cells were injected intraperitoneally in three groups of mice respectively (n = 5 in each group) (Fig. 7F). Tumor cells spread to several peritoneal organs 28 days after injection (Fig. 7F, right). The number of metastasis sites significantly increased in hTEM1 group (Fig. 7G, p < 0.01) and decreased in shRNA-TEM1 group (Fig. 7E, right, p < 0.05).

Collectively, the in vivo data suggested that TEM1 promotes the metastasis of uterine sarcoma cells without affecting tumor proliferation.

Taken together, these assays demonstrated the TEM1-mediated invasion and migration at two-dimensional and three-dimensional levels on gelatin matrix in MES-SA cells and in vivo xenografts. They also demonstrated the critical role of MMP-2 in TEM1-mediated ECM degradation.

## Discussion

### MMP-2, not MMP-9, might participate in the TEM1-mediated progression of uterine leiomyosarcoma

TEM1 has been considered a potential therapeutic target for sarcoma in the last decade [7, 44, 45]. Ontuxizumab (MORAb-004), has recently been applied as an effective humanized anti-TEM-1 monoclonal antibody in preclinical and clinical studies [11, 12]. However, to date, the role of TEM1 in tumor progression has not been clarified sufficiently. Yumiko Kondo et al. [46] suggested that TEM1 might be associated with tumor progression and metastasis in osteosarcoma and MORAb-004 could inhibit tumor cell migration in vitro. Consistent with our previous work [15] in which we found that TEM1 was overexpressed in uterine sarcoma specimens and promoted the migration and invasion of uterine sarcoma MES-SA cells, our present data showed that TEM1 expression level was correlated with tumor stage and MMP-2 expression. Leiomyoma specimens were included to allow comparison with its stromal origin as a benign tumor. TEM1 was highly expressed in leiomyosarcoma cells and correlated with tumor stage but not or weakly expressed in leiomyoma cells, implying that TEM1 might be a reliable and specific marker for tumor malignancy.

TEM1 and MMPs were reported to be co-expressed in tumor models. Margarida Maia et al. [19] reported a decrease in activated MMP-9 release in mice lacking the TEM1 cytoplasmic domain. Brian Tomkowicz [6] confirmed that MMP-9 activity was enhanced in generated CHO-TEM1 cells stably expressing TEM1 compared with wide-type CHO-K1 cells. However, our IHC results showed no correlation between TEM1 expression and MMP-9, but a positive correlation between TEM1 and MMP-2 in human uterine leiomyosarcoma specimens. Furthermore, we found that TEM1 and MMP-2 expression was positively correlated with tumor stage, implying that MMP-2, rather than MMP-9, might participate in TEM1-mediated tumorigenesis of uterine leiomyosarcoma.

MMP-2 has been reported to correlate with tumor metastasis in many cancer types. Yang Hyun Kee et al. [47] found significantly higher MMP-2 expression and slightly higher MMP-9 expression in metastatic malignant fibrous histiocytoma (MFH) compared with non-metastatic MFH (*P* < 0.05). Zhe Song [48] found a positive association of lymph nodes metastasis and the degree of lymphatic metastasis with the expression intensity of MMP-2 in hypopharyngeal carcinoma. Activated MMP-2 were highly expressed in breast cancers and were associated with invasive potential [49–51].

In our study, to explore the mechanism underlying the metastasis and invasion of uterine sarcoma, a poorly differentiated uterine sarcoma cell line MES-SA expressing moderate levels of TEM1 was used. We utilized these cells to establish TEM1 overexpression and knock-down in vitro models by lentivirus transfection. Our gelatinase zymography data displayed two bands corresponding to pro-MMP-2 and active-MMP-2 in MES-SA cells. However, MMP-9 bands were absent, which was consistent with previous findings by Roomi et al. [52] and our IHC staining results for MMP-2 and MMP-9 in uterine sarcoma specimens. The results confirmed the role of MMP-2 in promoting the uterine sarcoma progress.

### TEM1 promotes migration and invasion of MES-SA cells by upregulating MMP-2

MMP-2 regulation and mechanism of action is complex. Tissue inhibitor of metalloproteases 2 (TIMP-2) and MMP-14 are common modulators of MMP-2 [53–55]. Recent studies have shown that other molecules, including heparan sulfate [56], cytokines [57, 58], and estradiol [59] also modulate MMP-2 expression or activity.

MMP-2 can degrade ECM components. In an ovarian metastasis model, ECM components such as fibronectin, vitronectin and collagen I were cleaved into small fragments by activated MMP-2 and facilitate cancer cell adhesion and invasion by binding to integrin receptors [60]. TEM1, MMP-2 and collagen IV have been reported to co-exist in early angiogenesis, implying that TEM1 might interact with MMPs during angiogenesis and cell migration [6].

Our gain-of-function and loss-of-function experiments showed that high expression of TEM1 was accompanied by elevated secretion of activated MMP-2 followed by increased migration and invasion. Conversely, low expression of TEM1 is followed by lower expression level of MMP-2 and decreased aggressiveness of MES-SA cells. Downregulation of MMP-2 inhibited the tumorigenesis-promoting effect of TEM1. These results confirmed that MMP-2 up-regulated TEM1, further suggesting that TEM1 might promote the migration and invasion of uterine sarcoma through an MMP-2-dependent pathway.

### TEM1 promotes ECM remodeling by modulating MMP-2

Recent studies of TEM1 have been largely confined to promoting angiogenesis rather than ECM transformation [29, 39]. Robust TEM1-ECM interactions have been demonstrated in vivo by Brian Tomkowicz et al.’s [6] study [54]. They demonstrated that the extracellular region of TEM1 (Fc-TEM1) could directly bind to collagen I, IV, and fibronectin, but not to laminin and vitronectin. In contrast, we found that TEM1 promoted MES-SA cell adhesion to fibronectin, laminin and vitronectin. Moreover, we observed aggressive degradation activity of TEM1-overexpressing MES-SA cells on gelatin. A possible explanation for this observation is that malignant cells behave differently from non-malignant cells. Besides, other molecules, such as integrin, might also participate in modulating TEM1-ECM interactions in uterine sarcoma cells.

ECM degradation and remodeling caused by bioactive molecules such as MMPs released from cancer cells are crucial not only to normal development but also to tumor invasion and metastasis [42, 61, 62]. Invadopodia are podosome-like membrane protrusions formed by invasive malignant cells with matrix degradation ability [63]. Invadopodia were first described in breast cancer cell lines seeded on fibronectin-coated gelatin [64]. It has been demonstrated that MMPs, specially MMP-2, MMP-9 and MMP-13 are localized in invadopodia and play an important role in ECM proteolysis [65].

Mazurkiewicz E [41] described in her protocol that, dark spots or areas on a thin layer of fluorescent-labeled gelatin can be observed under fluorescence microscopy after cells are seeded in a given time, which is a result of active gelatin digesting by invadopodia. Invadopodia and focal degradation of gelatin matrix have been observed in PC-3 prostate cancer cells [66, 67], breast cancer MDA-MB-231 cells [40, 68, 69], and other malignant cell [70–72]. In the present study, gelatin degradation of MDA-MB-231 cells were found within the cell region, which was confirmed by literatures [3, 30, 39, 43]. However, aggressive gelatin degradation caused by hTEM1-MES-SA cells can cause pool-like degradation areas similar to studies of Albrechtsen R [40], possibly due to degradation on different layers and active cell migration. TEM1-overexpressed (hTEM1) MES-SA cell line displayed the highest percentage of degradation and invadopodia formation. Pool-like degradation areas were commonly seen on gelatin matrix in hTEM1 MES-SA cells, reflecting a tree-like migrating track or/and pool-like vertical invasion of cell behaviors. From three-dimensional videos, clear migrating routes of a single cell and different layers of gelatin-matrix degradation were revealed. Treatment with MMP-2 siRNA significantly impaired the ability of matrix degradation and invadopodia formation of the cells. Our results of migration and invasion transwell assay on matrix also confirmed that TEM1 promoted the migration and invasion ability of MES-SA cells, which was inhibited by MMP-2 knockdown.

### TEM1 promotes the metastasis of uterine sarcoma cells rather than tumor growth in vivo

It is clear that inhibition of TEM1 in cancer-associated fibroblasts significantly impaired the tumor growth of hepatocellular carcinoma [5] and lung cancer [73] in xenograft models. To evaluate the role of TEM1 in promoting uterine sarcoma proliferation and metastasis *in vivo*, we established MES-SA cells-bearing subcutaneous and intraperitoneal mice models. Our results showed that TEM1 promoted the metastasis of uterine sarcoma cells, while tumor proliferation was not affected. The MMP-2 mRNA expression was increased in hTEM1 subcutaneous tumors, which was in agreement of the MMP-2 up-regulation effect of TEM1 in vitro. The in vivo data validated the role of TEM1 in regulating MMP-2 and promoting metastasis in uterine sarcoma.

These findings suggested that TEM1-overexpressing MES-SA cells displayed an enhanced capacity to degrade ECM. The enhanced cell migration and invasion by TEM1 overexpression might be the result of ECM remodeling upregulated by MMP-2.

## Conclusion

Our analysis revealed a high expression pattern of TEM1 and MMP-2 in uterine leiomyosarcoma with percentage of 92% (23/25) and 88% (22/24). TEM1 and MMP-2 were highly expressed in all 17 of high stage (III-IV) uterine sarcoma specimens. TEM1 promoted uterine sarcoma migration and invasion via increasing MMP-2 activity and ECM remodeling/degradation. The above provides valuable insights into a potential TEM1-targeted therapy for uterine sarcoma.

## Supplementary Information

Supplementary file 1 (pptx 56 KB)

## Data Availability

The original contributions presented in the study are included in the article/supplementary file. Further inquiries can be directed to the corresponding author.
