# Supplementary material for: TEM1 up-regulates MMP-2 and promotes ECM remodeling for facilitating invasion and migration of uterine sarcoma
Source: Discov Oncol. 2023 Jan 13;14:5. doi: 10.1007/s12672-023-00613-6 (PMC9839929; doi:10.1007/s12672-023-00613-6)
Supplement: Supplementary file 1 — (pptx 56 KB) [file 12672_2023_613_MOESM1_ESM.pptx]

## Slide 1
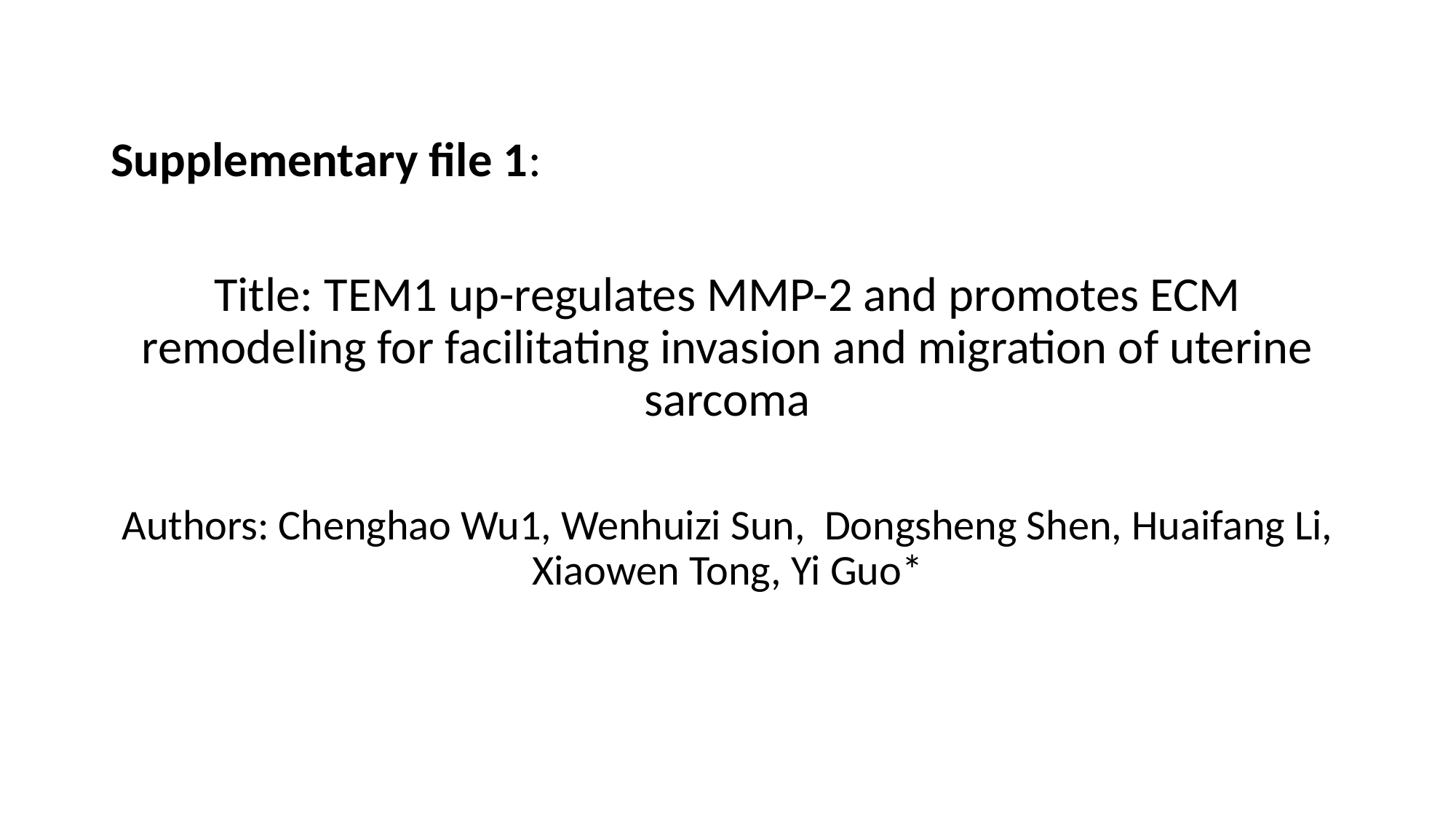

Supplementary file 1:
Title: TEM1 up-regulates MMP-2 and promotes ECM remodeling for facilitating invasion and migration of uterine sarcoma
Authors: Chenghao Wu1, Wenhuizi Sun, Dongsheng Shen, Huaifang Li, Xiaowen Tong, Yi Guo*

## Slide 2
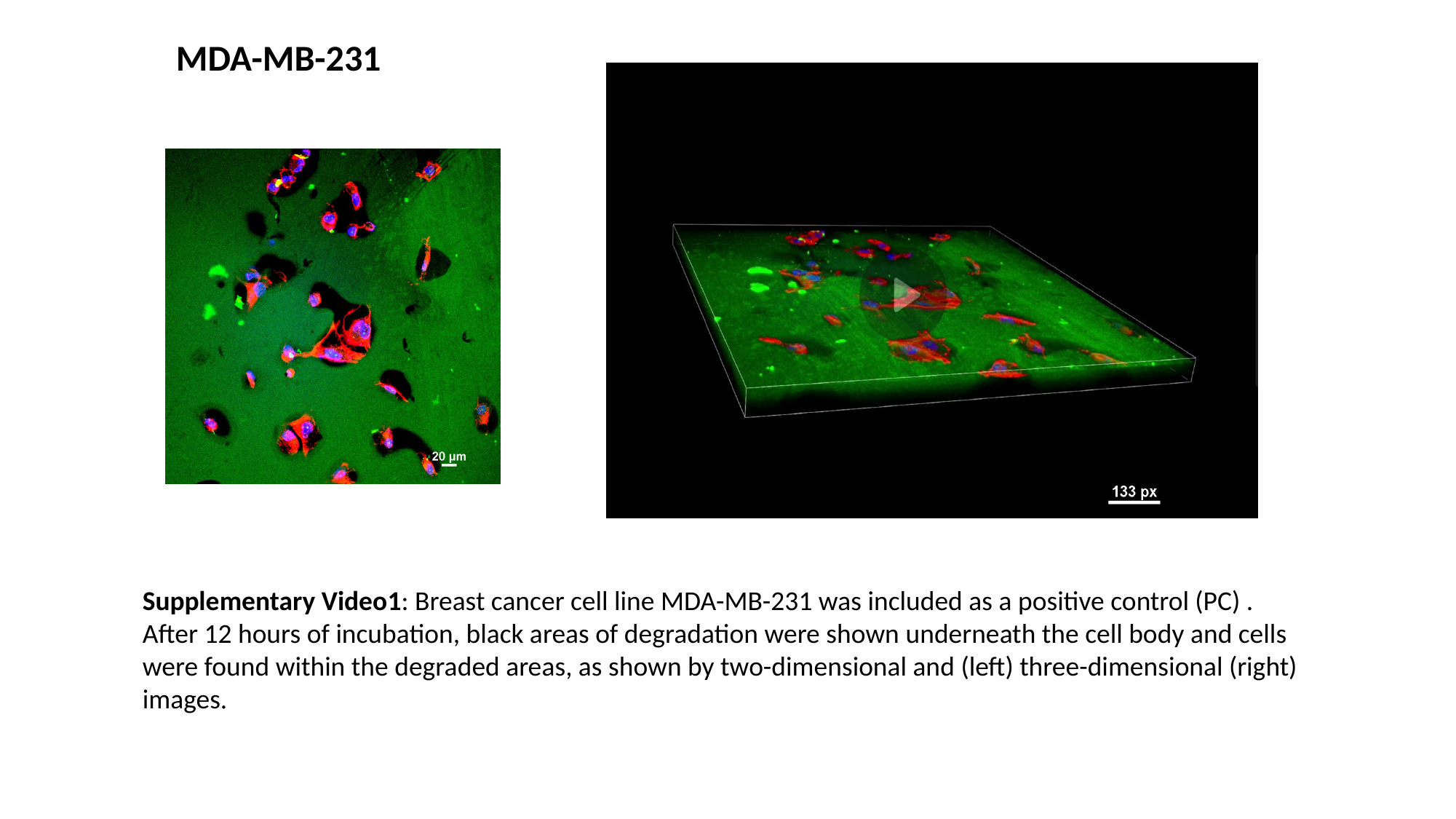

MDA-MB-231
Supplementary Video1: Breast cancer cell line MDA-MB-231 was included as a positive control (PC) . After 12 hours of incubation, black areas of degradation were shown underneath the cell body and cells were found within the degraded areas, as shown by two-dimensional and (left) three-dimensional (right) images.

## Slide 3
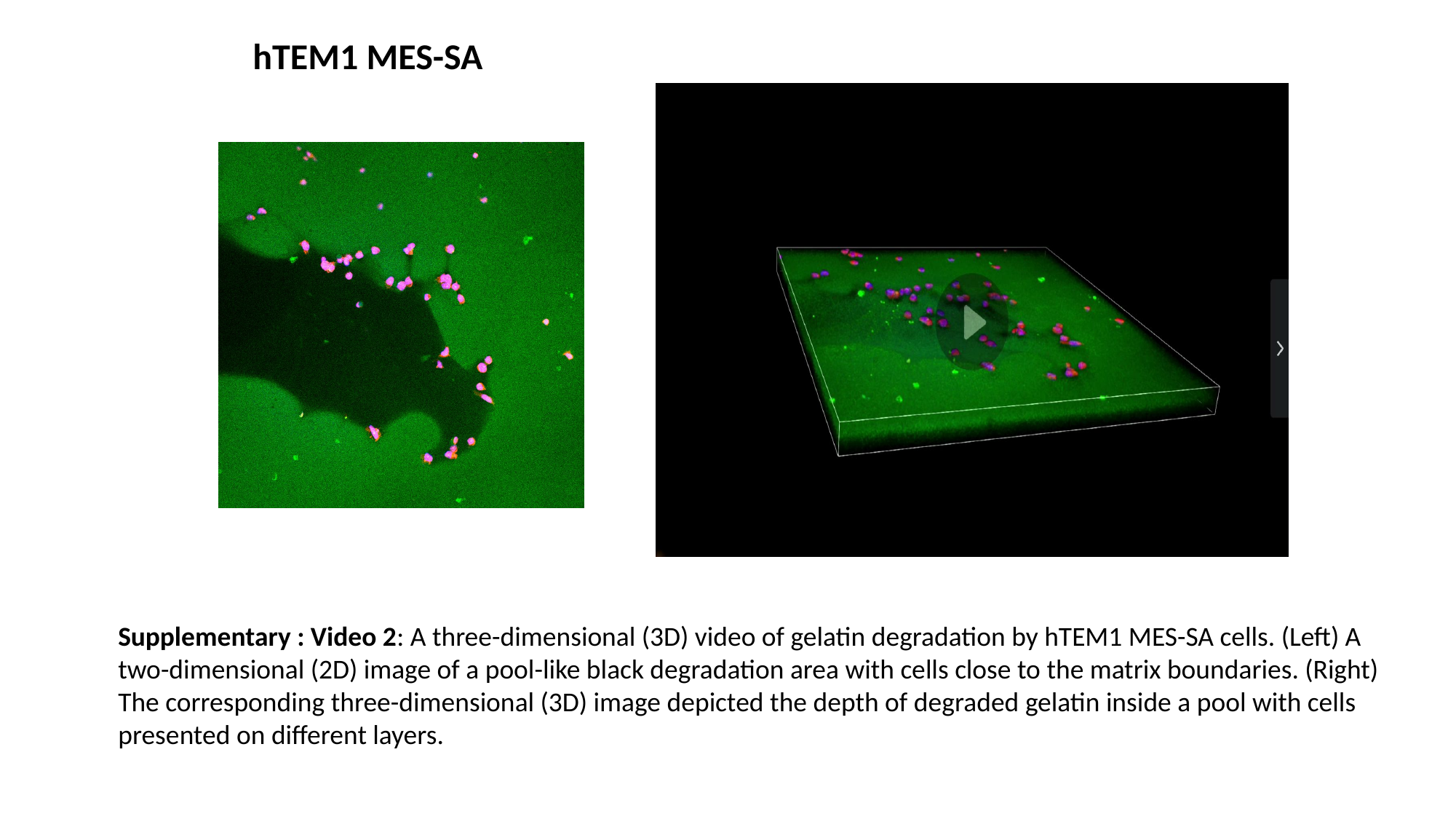

hTEM1 MES-SA
Supplementary : Video 2: A three-dimensional (3D) video of gelatin degradation by hTEM1 MES-SA cells. (Left) A two-dimensional (2D) image of a pool-like black degradation area with cells close to the matrix boundaries. (Right) The corresponding three-dimensional (3D) image depicted the depth of degraded gelatin inside a pool with cells presented on different layers.

## Slide 4
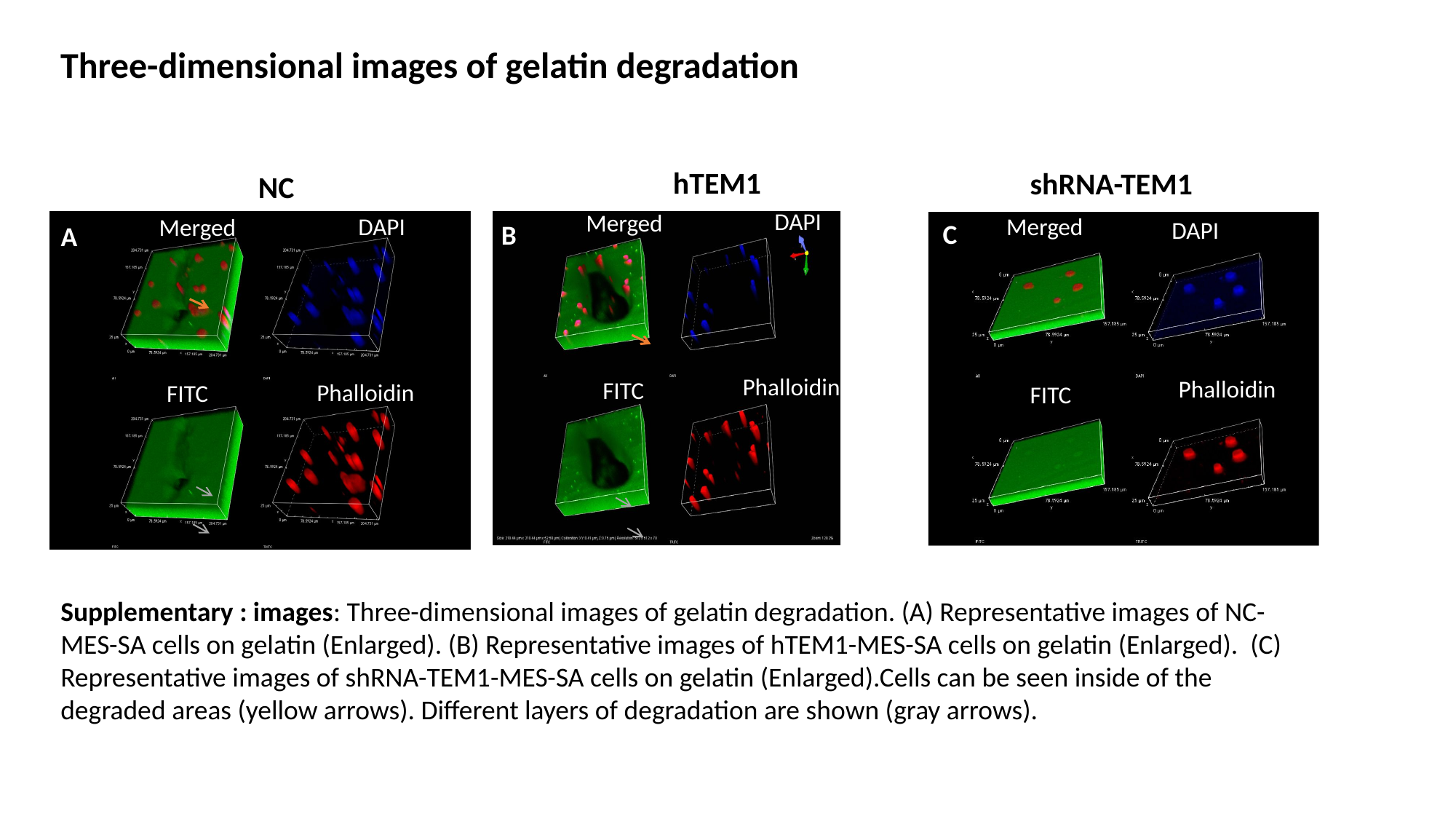

# Three-dimensional images of gelatin degradation
hTEM1
shRNA-TEM1
NC
DAPI
Merged
Merged
DAPI
Merged
DAPI
C
B
A
Merged
Phalloidin
Phalloidin
FITC
Phalloidin
FITC
FITC
Supplementary : images: Three-dimensional images of gelatin degradation. (A) Representative images of NC-MES-SA cells on gelatin (Enlarged). (B) Representative images of hTEM1-MES-SA cells on gelatin (Enlarged). (C) Representative images of shRNA-TEM1-MES-SA cells on gelatin (Enlarged).Cells can be seen inside of the degraded areas (yellow arrows). Different layers of degradation are shown (gray arrows).
